# Supplementary material for: Maximum Tolerated Dose and Anti-Tumor Activity of Intraperitoneal Cantrixil (TRX-E-002-1) in Patients with Persistent or Recurrent Ovarian Cancer, Fallopian Tube Cancer, or Primary Peritoneal Cancer: Phase I Study Results
Source: Cancers (Basel). 2021 Jun 26;13(13):3196. doi: 10.3390/cancers13133196 (PMC8268018; doi:10.3390/cancers13133196)
Supplement: Supplementary file 1 [file cancers-13-03196-s001.zip › cancers-1261175-supplementary.pdf]

Supplementary Data

Coward JI et al. Phase I Study of Intra-peritoneal Cantrixil (TRX-E-002-1) in Patients With Persistent or Recurrent Ovarian Cancer, Fallopian Tube Cancer or Primary Peritoneal Cancer

Supplementary File A.

Summary of Methods

Permitted Chemotherapy Combination Therapies

Regimen	Reference
Carboplatin AUC 4 over 1 hour on Day 4 every 21 days for 6 cycles	Dizon et al., 2015
Liposomal doxorubicin 40-50 mg/m^2 intravenously over 30 min on Day 4 every 21 days	Gordon et al., 2004
Gemcitabine 1000 mg/m^2 intravenously over 30 min on Days 4 and 11 (Parts A and B); every 21 days	Markman et al., 2003
Paclitaxel 80 mg/m^2 intravenously over 1 hour weekly on Days 4, 11, and 18 (Parts A and B)	Markman et al., 2006
Docetaxel 75-100 mg/m^2 intravenously over 1 hour on Day 4 every 21 days	Rose et al., 2003
Topotecan 4.0 mg/m^2 on Days 4, 11, and 18 every 21 days	Sehouli et al. 2011
Best supportive care and extended monotherapy with Cantrixil alone	

AUC: Area under the plasma concentration-time curve; CSP: Clinical Study Protocol.

PART A: Dose escalation schedule

Patients were started at Dose Level 0, which was calculated as the human equivalent of 10% of the severely toxic dose in 10% of animals tested (STD10) dose in rodents. Single patient cohorts were treated with increasing doses of Cantrixil until an AE was observed during Cycle 1 that met the definition of a DLT or, in the opinion of the DSMB and the Investigator, warranted observing additional patients at this dose level; at this point the study was to revert to a 3+3 rules-based dose escalation study. The decision to expand from single patient cohorts to 3 patient cohorts was critical for the safety of participants and was made with input from the Investigator and DSMB responsible for the study. Dose Levels -1 and -2 were only activated if there were 2 DLTs at Dose Level 0 and -1, respectively. Once the study entered a 3+3 rules-based design, the study was not reverted back to single patient cohorts.

Dose Level	Cantrixil	Patient Number
Level -2	0.06 mg/kg<br>Dose is half of Level -1	n = 2 to 6
Level -1	0.12 mg/kg<br>Dose is half of Level 0	n = 2 to 6
Starting dose:<br>Level 0	0.24 mg/kg<br>Dose is equivalent to 10% of STD10	n = 1 to 6
Level 1	0.6 mg/kg<br>Dose is 2.5 × Dose Level 0	n = 1 to 6
Level 2	1.25 mg/kg<br>Dose is 2.1 × Dose Level 1	n = 1 to 6
Level 3	2.5 mg/kg<br>Dose is 2 × Dose Level 2	n = 1 to 6
Level 4	5.0 mg/kg<br>Dose is 2 × Dose Level 3	n = 1 to 6
Level 5	10.0 mg/kg<br>Dose is 2 × Dose Level 4	n = 1 to 6
Level 6	20.0 mg/kg<br>Maximum dose to test	n = 1 to 6

STD10: Severely toxic dose in 10% of animals tested.

3+3 Design Dose escalation Plan

Number of patients with DLT at a given dose level	Action
0 out of 3	Enter at least 3 patients at the next dose level.
1 out of 3	Enter at least 3 more patients at this dose level and<br>*if 0 of these 3 new patients experienced a DLT*, proceed to the next dose level;<br>*if ≥1 of this group suffer a DLT (for a total of ≥2/6 patients with a DLT)*, this dose exceeded the MTD and dose escalation was stopped. To further assess tolerability, 3 additional patients were entered at the next lowest dose level if only 3 patients were treated previously at that dose. Upon determination of the MTD, the study was to proceed to Part B.
≥2	Dose escalation were stopped. This dose exceeded the MTD. To further assess tolerability, 3 additional patients were entered at the next lowest dose level if only 3 patients were treated previously at that dose and the study was to proceed to Part B.

DLT: Dose-limiting toxicity; MTD: Maximum tolerated dose.

Definitions of unacceptable adverse events or dose limiting toxicities

If any of the following unacceptable AEs or DLTs were observed and unless clearly unrelated to study treatment (eg, disease progression), treatment at the currently allocated Cantrixil dose was paused. At this point a dose reduction may have been considered or the treatment may have been terminated, depending on what was considered by the Investigator to be in the participant's best interest:

- Haematologic toxicity:
  - Grade 4 neutropenia, lasting at least 5 days,
  - Grade 3 or Grade 4 neutropenia associated with fever >38.5°C,
  - Grade 4 thrombocytopenia lasting at least 5 days,
  - Grade 3 thrombocytopenia associated with severe bleeding in the opinion of the Investigator,
  - Dose delay of ≥3 weeks due to failure to recover counts to levels described in the study inclusion criteria.
- Any Common Terminology Criteria for Adverse Events (CTCAE) Version 4.03 Grade 3 or Grade 4 nonhematological toxicity except:
  - Alopecia,
  - Grade 3 abdominal pain deemed related to the port or catheter as determined by the treating physician,
  - Grade 3 anorexia,
  - Grade 3 fatigue,
  - Grade 3 nausea and/or vomiting, or diarrhoea, lasting ≤48 hours with or without maximal medical management,
  - Grade 3 dehydration because of nausea and vomiting,
  - Grade 3 constipation,
  - Grade 3 metabolic abnormalities (hypokalaemia, hypomagnesaemia, hypocalcaemia, hypophosphatemia) that recovers to Grade 1 or less within 48 hours with or without medical management.
- Other SAEs, which in the opinion of the treating Investigator, necessitate temporary or permanent cessation of administration.
- Treatment delays of ≥3 weeks due to any nonhematological toxicity were to constitute a DLT.

Supplementary Figure S1.

Pharmacokinetic Data versus Dose

Individual dose-normalised pharmacokinetic parameters after administration of Cantrixil on Cycle 1, Day 1, Cycle 3, Day 1 & Cycle 3, Day 8 in Part A and Part B, as a function of dose.

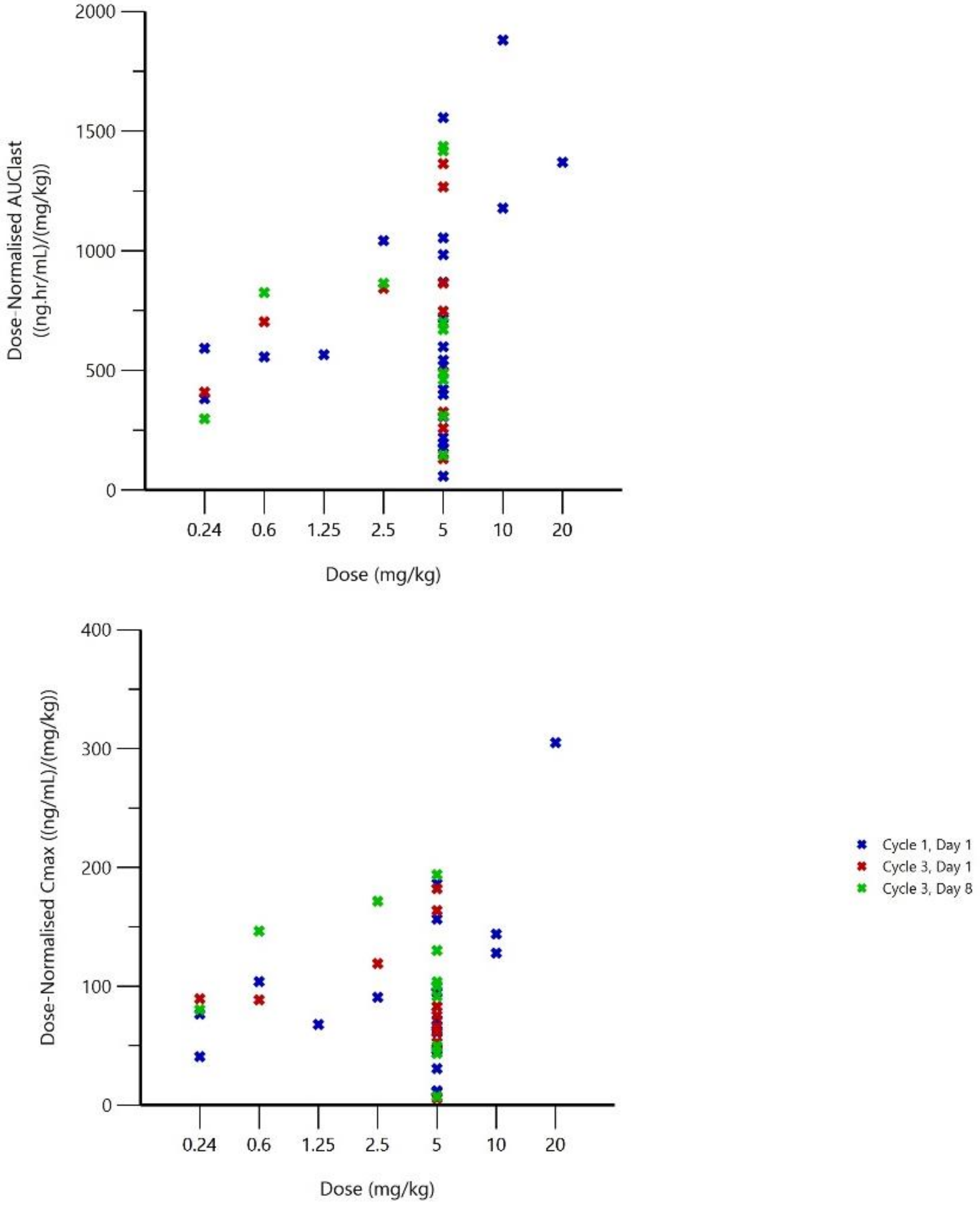

Supplementary Figure S2.

Patient Factors vs Dose-Normalised Pharmacokinetic Data (mg): BMI

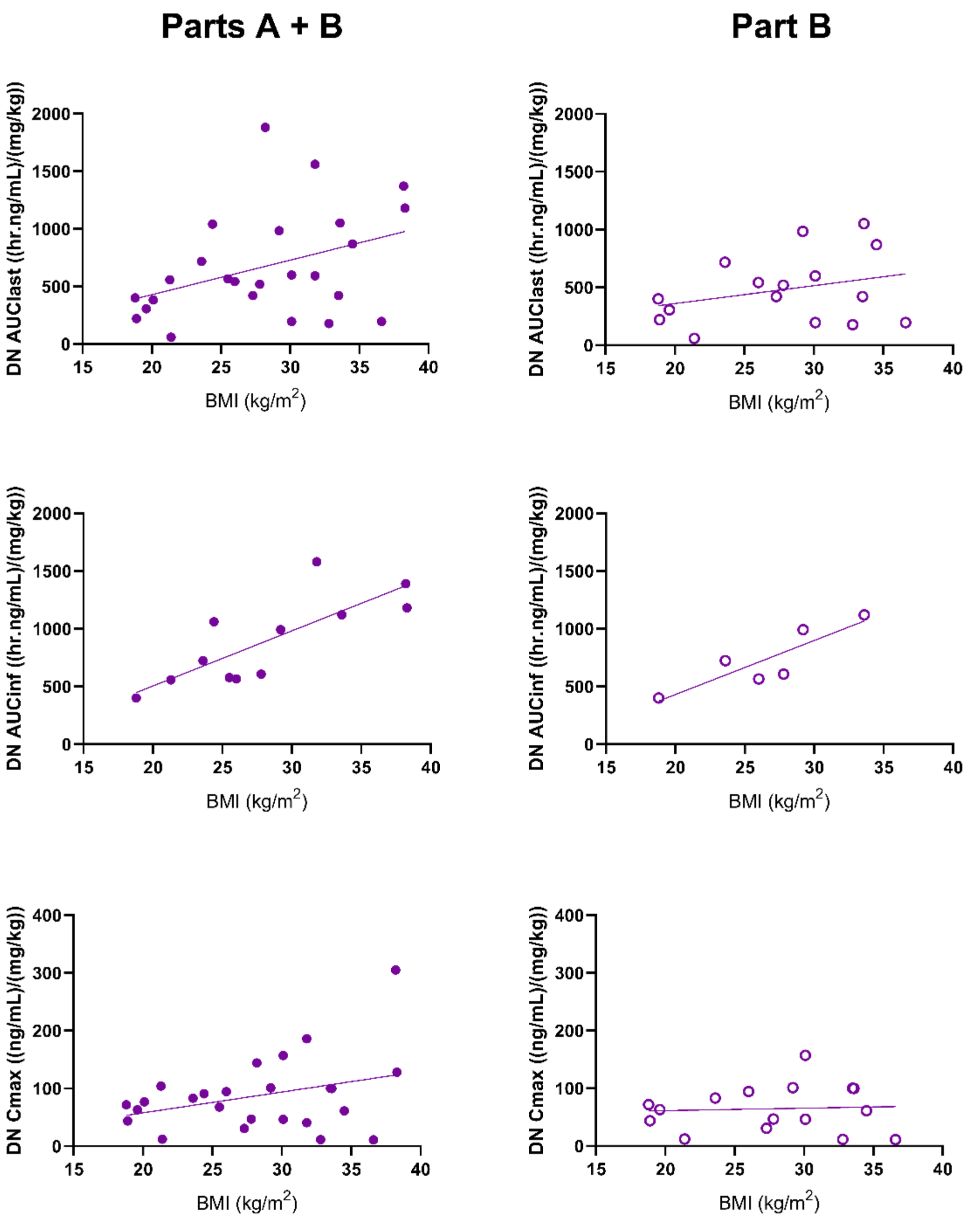

Supplementary Table S1.

Summary of All Treatment-Emergent Treatment-Related Adverse Events in ≥3 Patients of Overall Patients by System Organ Class and Preferred Term - Safety Population - Part A (All Doses), Patients Receiving MTD, and Overall Patients

		Monotherapy			Combination therapy			Overall		
**System Organ Class/ MedDRA Preferred Term**	**Statistic**	**Part A All Doses (N=11)**	**MTD 5.0 mg/kg (N=17)**	**Overall Patients (N=25)**	**Part A All Doses (N=11)**	**MTD 5.0 mg/kg (N=17)**	**Overall Patients (N=25)**	**Part A All Doses (N=11)**	**MTD 5.0 mg/kg (N=17)**	**Overall Patients (N=25)**
Number of patients with any treatment-related TEAEs	n(%)	9(81.8)	12(70.6)	18(72.0)	3(27.3)	4(23.5)	5(20.0)	10(90.9)	13(76.5)	20(80.0)
Gastrointestinal disorders	n(%)	8(72.7)	11(64.7)	16(64.0)	2(18.2)	3(17.6)	4(16.0)	9(81.8)	12(70.6)	18(72.0)
Abdominal pain	n(%)	7(63.6)	5(29.4)	10(40.0)	2(18.2)	2(11.8)	3(12.0)	8(72.7)	6(35.3)	12(48.0)
Vomiting	n(%)	5(45.5)	7(41.2)	10(40.0)	-	-	-	5(45.5)	7(41.2)	10(40.0)
Nausea	n(%)	5(45.5)	4(23.5)	7(28.0)	-	-	-	5(45.5)	4(23.5)	7(28.0)
Abdominal distension	n(%)	2(18.2)	3(17.6)	4(16.0)	-	-	-	2(18.2)	3(17.6)	4(16.0)
Diarrhoea	n(%)	3(27.3)	2(11.8)	4(16.0)	-	-	-	3(27.3)	2(11.8)	4(16.0)
Abdominal discomfort	n(%)	1(9.1)	1(5.9)	2(8.0)	1(9.1)	0(0.0)	1(4.0)	2(18.2)	1(5.9)	3(12.0)
General disorders and administration site conditions	n(%)	6(54.5)	3(17.6)	8(32.0)	2(18.2)	2(11.8)	3(12.0)	8(72.7)	5(29.4)	11(44.0)
Fatigue	n(%)	5(45.5)	3(17.6)	7(28.0)	2(18.2)	1(5.9)	2(8.0)	7(63.6)	4(23.5)	9(36.0)
Investigations	(%)	1(9.1)	1(5.9)	2(8.0)	-	1(5.9)	1(4.0)	1(9.1)	2(11.8)	3(12.0)

AE: Adverse event; CRF: Case report file; MedDRA: Medical Dictionary for Regulatory Activities; MTD: Maximum tolerated dose; TEAE: Treatment-emergent adverse event.
A patient experiencing multiple occurrences of an adverse event was counted, at most, once per system organ class and preferred term.
The numbers of patients within each column cannot be added because a patient may have had more than 1 adverse event.
System organ class and preferred term are from the MedDRA dictionary version 20.0.
Related TEAEs are defined as those responded as 'Yes' or have missing response to question relationship to study drug in CRF.
Adverse events started after first Cantrixil dosing till first chemotherapy agent are considered under monotherapy. Adverse events started after first chemotherapy are considered under combination therapy. Overall includes all AEs in monotherapy and combination.
All percentages are based on number of patients in Safety Population.
Table includes patients from Parts A and B receiving MTD for 5.0 mg/kg group and patients from both parts together for Overall group.
Source: Table 14.3.1.5.1 and Table 14.3.1.5.2.

Supplementary Table S2.
Exploratory analysis of stem cell markers

(Population: Part A only)

Timepoint	ALDH (%)	CD44 (%)	CETC (cells/ml)	Spheroids (%)
**Screening, n**	**3**	**1**	**9**	**9**
Mean, SD	943.7 (1477.76)	95.0 (-)	105.6 (186.15)	413.7 (866.58)
Median (Min,Max)	100 (81,2650)	95 (95,95)	0 (0,550)	0 (0,2650)
**Week 6, n**	**2**	**2**	**5**	**5**
Mean, SD	80.0 (28.2)	87.5 (17.68)	640.0 (879.2)	140 (258.36)
Median (Min,Max)	80 (60,100)	87.5 (75,100)	300 (100,2200)	50 (0,600)
**End of therapy, n**	**-**	**-**	**5**	**5**
Mean, SD	-	-	170 (125.5)	0 (0.0)
Median (Min,Max)	-	-	150 (0,350)	0 (0, 0)

ALDH, Aldehyde Dehydrogenase; CD44, Ovarian Cancer Stem-like Cells; CETC, Circulating Epithelial Tumour Cells; Max, maximum, Min, minimum; SD, standard deviation.
Week 6 = end of cycle 2, week 12 = end of cycle 4, week 16 = end of cycle 6, week 24 = end of cycle 8.
